# gga-miR-451 Negatively Regulates *Mycoplasma gallisepticum* (HS Strain)-Induced Inflammatory Cytokine Production via Targeting YWHAZ

**DOI:** 10.3390/ijms19041191

**Published:** 2018-04-13

**Authors:** Yabo Zhao, Kang Zhang, Mengyun Zou, Yingfei Sun, Xiuli Peng

**Affiliations:** Key Laboratory of Agricultural Animal Genetics, Breeding and Reproduction (Huazhong Agricultural University), Ministry of Education, College of Animal Science and Technology and College of Veterinary Medicine, Huazhong Agricultural University, Wuhan 430070, China; zyb@webmail.hzau.edu.cn (Y.Z.); zhangkang123@webmail.hzau.edu.cn (K.Z.); zoumengyun@webmail.hzau.edu.cn (M.Z.); sunyingfei@webmail.hzau.edu.cn (Y.S.)

**Keywords:** *Mycoplasma gallisepticum* HS strain, gga-miR-451, YWHAZ, inflammatory cytokines

## Abstract

*Mycoplasma gallisepticum* (MG) is the most economically significant mycoplasma pathogen of poultry that causes chronic respiratory disease (CRD) in chickens. Although miRNAs have been identified as a major regulator effect on inflammatory response, it is largely unclear how they regulate MG-induced inflammation. The aim of this study was to investigate the functional roles of gga-miR-451 and identify downstream targets regulated by gga-miR-451 in MG infection of chicken. We found that the expression of gga-miR-451 was significantly up-regulated during MG infection of chicken embryo fibroblast cells (DF-1) and chicken embryonic lungs. Overexpression of gga-miR-451 decreased the MG-induced inflammatory cytokine production, including tumor necrosis factor-α (TNF-α), interleukin-1β (IL-1β), and interleukin-6 (IL-6), whereas inhibition of gga-miR-451 had the opposite effect. Gene expression data combined with luciferase reporter assays demonstrated that tyrosine3-monooxygenase/tryptophan5-monooxygenase activation protein zeta (YWHAZ) was identified as a direct target of gga-miR-451 in the context of MG infection. Furthermore, upregulation of gga-miR-451 significantly inhibited the MG-infected DF-1 cells proliferation, induced cell-cycle arrest, and promoted apoptosis. Collectively, our results demonstrate that gga-miR-451 negatively regulates the MG-induced production of inflammatory cytokines via targeting YWHAZ, inhibits the cell cycle progression and cell proliferation, and promotes cell apoptosis. This study provides a better understanding of the molecular mechanisms of MG infection.

## 1. Introduction

The host inflammatory response constitutes an essential immune defense against invasion by microbial pathogens. It is a protective process to clear the detrimental invaders. Nevertheless, an excessive inflammatory response to overwhelm pathogens can be fatal [1]. *Mycoplasma gallisepticum* (MG) is a common etiological cause of chronic respiratory disease (CRD) in chickens and infectious sinusitis in turkeys [2], which feature inflammation in respiratory tract (trachea, lungs, and air sacs) [3,4]. Controlling the impact of the disease on a global level is done by eradication of positive breeder flocks or by vaccination and medication; it is impossible to completely avoid the influence of MG infection. As a consequence, MG continues to cause enormous economic losses in the form of drop in egg production, poor hatchability, reduced weight gain, the downgrading of the carcass, and decreased feed conversion ratio [5,6]. MG also can invade, survive, and multiply inside chicken embryonic fibroblasts (CEF) and HeLa cells in vitro [7,8,9]. During infection, MG interacts with host respiratory epithelial cells and generates an inflammatory response, resulting in increased levels of cytokines, such as tumor necrosis factor alpha (TNF-α), interleukin-6 (IL-6), and interleukin-2 (IL-2) [10]. The increased levels of inflammatory mediators appear to play a protective role or to initiate an irreversible immune response leading to cell death [11]. However, the regulation of MG-induced respiratory inflammation is not well documented. MG-HS strain is a virulence strain isolated from a chicken farm in Hubei Province of China, which is used for further experiments [12,13].

Microribonucleic acids (miRNA) are important post-transcriptional regulators in almost all biological processes but their roles within avian inflammatory disease have not been well characterized. These small non-coding RNAs negatively regulate protein levels by interacting with target mRNAs via partial or full sequence complementarity, which triggers mRNA degradation or blocks translation [14,15]. miRNAs can act as fine-tuners to switch the levels of translatable mRNA, to decrease protein production via maintaining mRNA levels below a threshold [16]. Fine-tuning of protein levels by miRNAs has been shown to modulate developmental programs, innate and adaptive immunity, and cellular responses to infection [4,17,18,19,20]. Accumulating evidence also indicates a decisive role of miRNAs in inflammatory responses. For instance, miR-155 modulates inflammatory cytokine production in human dendritic cells while lipopolysaccharide stimulates these cells [21]. miR-21 and miR-146a are deemed as regulators of nuclear factor kappa B (NF-κB) signaling and inflammatory responses at multiple levels [22,23]. Other miRNAs, including miR-16 and miR-29a, are reported to participate in the protumoral inflammatory process by activating the TLR8 response on immune cells [15]. Recently, we also reported the role of gga-miR-101 and gga-miR-19a in regulating MG-HS infection and MG-HS-mediated inflammatory cytokine production in both DF-1 cells and the lungs of chicken embryos [24,25].

miR-451 has been reported to be induced in influenza-infected cells and as a key factor involved in regulating inflammation [26]. Other researchers have shown that miR-451 regulates the expression of tyrosine3-monooxygenase/tryptophan 5-monooxygenase activation protein, zeta (YWHAZ/14-3-3ζ) by binding to the 3′ untranslated region (3′-UTR) of the YWHAZ and that miR-451 plays an essential role in a number of disease processes [27,28,29,30]. However, the role of gga-miR-451 in MG-infected chickens has not been reported.

In the present study, we found that gga-miR-451 is significantly up-regulated in MG-infected chicken embryonic lungs and DF-1 cells and is a negative regulator of inflammatory cytokine production. Further investigation revealed that YWHAZ is a target gene of gga-miR-451; gga-miR-451 inhibits MG-infected DF-1 cell proliferation and the cell cycle progression, and induces cell apoptosis.

## 2. Results

### 2.1. MG Infection Significantly Upregulates gga-miR-451 Expression

**Figure 1:**
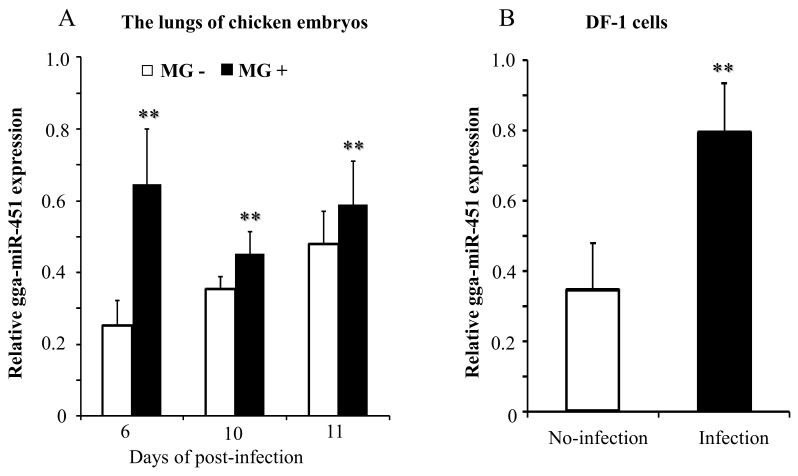
gga-miR-451 expression is upregulated after *Mycoplasma gallisepticum* (MG) infection in the lungs of chicken embryos (**A**) and DF-1 cells (**B**). The total RNA was extracted, and gga-miR-451 expression was assessed by RT-qPCR using 5S-rRNA as the internal quantitative control gene. Three independent experiments, each with three replicates, were performed. Student *t* test. The plotted data points show the means ± SDs, and the asterisks indicate statistically significant differences (** *p* < 0.01).

miRNAs sequencing was performed previously and a large variety of dysregulated miRNAs were identified in the lungs of MG-infected chicken embryos, and gga-miR-451 was down-regulated during MG infection [31]. To verify this result, chicken embryos were infected with MG-HS on the ninth day of incubation. On days 6, 10, and 11 post-infection (equivalent to days 15, 19, and 20 of egg incubation), the gga-miR-451 levels were determined by quantitative real-time PCR (qRT-PCR). The data showed that gga-miR-451 expression was significantly up-regulated in the lungs of MG-infected chicken embryos when compared to non-infected lungs (Figure 1A). This result was opposite to our miRNAs sequencing results. Whereupon, we further investigated the expression of gga-miR-451 in MG-infected DF-1 cells at 48 h post-infection, and the result demonstrated that gga-miR-451 expression was up-regulated after MG infection (Figure 1B). Taken together, these results indicate that gga-miR-451 level is significantly elevated during MG infection.

### 2.2. gga-miR-451 Reduces MG–Mediated Inflammatory Cytokine Production

**Figure 2:**
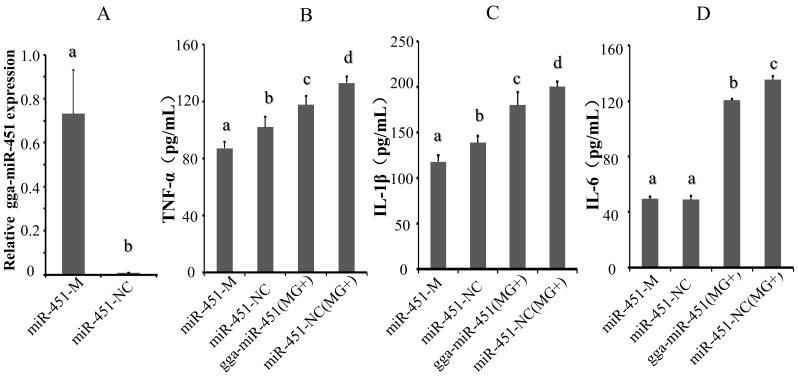

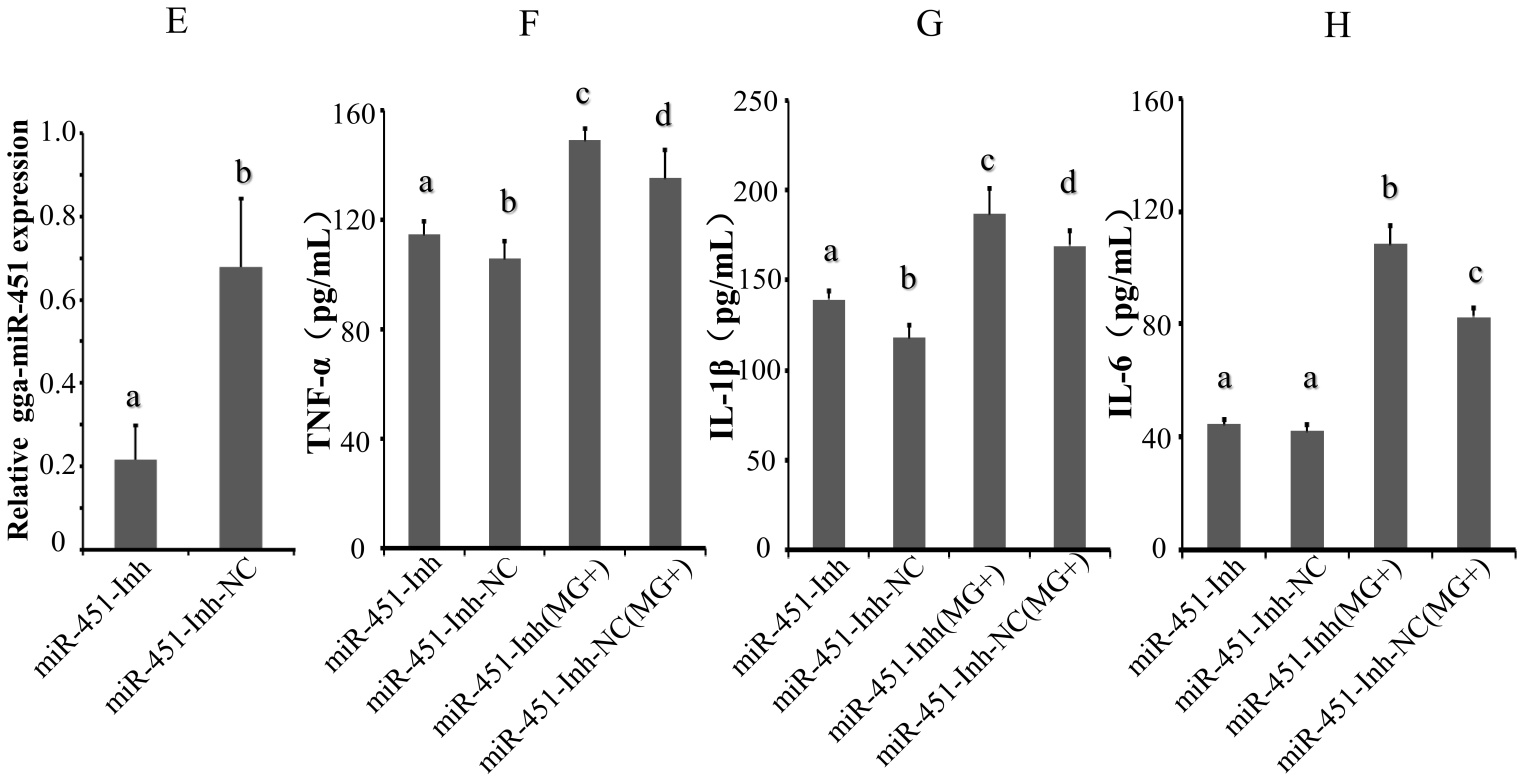
gga-miR-451 reduces MG-HS-mediated production of inflammatory cytokines. (**A**) Over-expressing miR-451-M (gga-miR-451 mimics) increases the expression of gga-miR-451 in DF-1 cells. (**E**) Transfection of miR-451-Inh (gga-miR-451 Inhibitor) reduces the expression of gga-miR-451 in DF-1 cells. gga-miR-451 expression was assessed by RT-qPCR using 5S-rRNA as the internal quantitative control gene. (**B**–**D**, **F**–**H**) DF-1 cells were transfected with miR-451-M, miR-451-NC, miR-451-Inh, or miR-451-Inh-NC and were incubated for 24 h and then either left uninfected or infected with 130 µL of MG-HS for 36 h (denoted as miR-451-M (MG+), miR-451-NC (MG+), miR-451-Inh (MG+), and miR-451-Inh-NC (MG+), respectively). The protein levels of TNF-α, IL-1β, and IL6 were analyzed by ELISA at 36 h post-infection. Three independent experiments, each with three replicates, were performed. The data are presented as the means ± SDs. One-way ANOVA was used to analyze significant differences (Different lowercase letters represent *p* < 0.01).

The MG-HS infection results in host immune response along with the production of inflammatory cytokines [10,24]. To examine whether gga-miR-451 is involved in the MG-HS-mediated inflammatory process, inflammatory cytokine levels were determined by enzyme-linked immunosorbent assay (ELISA). First, we evaluated the quality of synthetic gga-miR-451 mimics and inhibitor on the expression pattern of gga-miR-451. miRNA mimics are double-stranded RNAs synthesized to simulate naturally occurring mature miRNAs, whereas the inhibitor are chemically modified antisense single-stranded RNAs that silence the endogenous miRNAs by sequence complementarity. miRNA mimics and inhibitor can cause changes in the levels of the physiological miRNAs. We transfected DF-1 cells with gga-miR-451 mimics (denoted as miR-451-M), a gga-miR-451-Negative Control (denoted as miR-451-NC), a gga-miR-451 inhibitor (denoted as miR-451-Inh), or a random miRNA inhibitor (denoted as miR-451-Inh-NC), respectively. As expected, transfection of gga-miR-451 mimics increased gga-miR-451 levels significantly in DF-1 cells at 48 h post-transfection (Figure 2A), whereas gga-miR-451 inhibitor diminished its levels (Figure 2E). DF-1 cells were transfected with miR-451-M, miR-451-NC, miR-451-Inh, or miR-451-Inh-NC for 24 h, and were then infected with 130 µL of MG-HS strain at 10^10^ CCU/mL (denoted as miR-451-M (MG+), miR-451-NC (MG+), miR-451-Inh (MG+), and miR-451-Inh-NC (MG+), respectively) for 36 h. Compared with miR-451-M and NC groups, gga-miR-451 mimics significantly down-regulated the protein levels of TNF-α, IL-1β, and IL6 in MG-infected DF-1 cells (Figure 2B–D). In contrast, the inhibition of endogenous gga-miR-451 significantly increased the protein levels of TNF-α and IL-1β (Figure 2F–H). IL6 expression was consistent with TNF-α and IL-1β in MG-infected DF-1 cells, however, IL6 expression was not effected by gga-miR-451 in non-infected DF-1 cells. Thus, these data strongly indicate that gga-miR-451 reduces the production of inflammatory cytokines in MG-HS-infected cells.

### 2.3. YWHAZ Is a Direct Target of gga-miR-451

**Figure 3:**
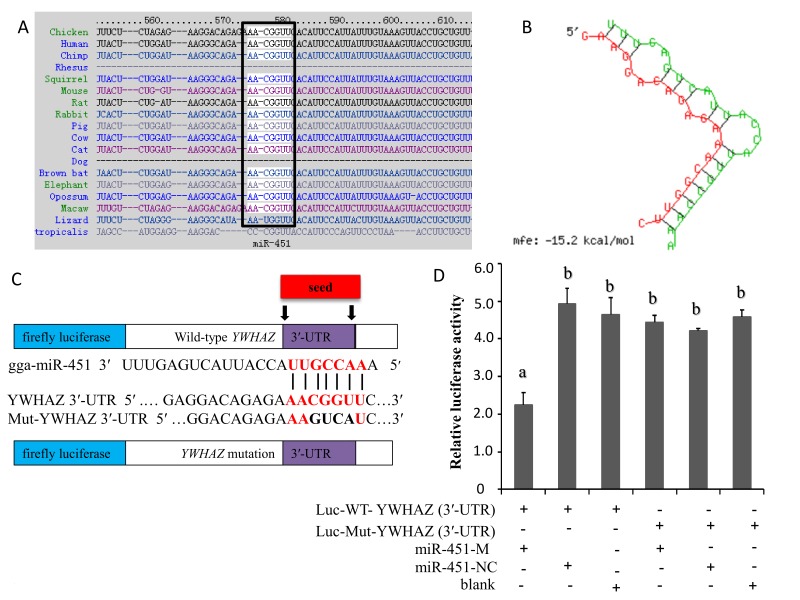
YWHAZ is a direct target of gga-miR-451. (**A**) Sequence alignment of YWHAZ 3′-UTR from different species. The conserved target sequences are highlighted. (**B**) The secondary structure of the RNA duplex of gga-miR-451 and the YWHAZ 3′-UTR target site (red: YWHAZ sequence; green: gga-miR-451). (**C**) psiCHECK-2 dual-luciferase reporter vector containing the 3′-UTR (wild-type or mutant) of YWHAZ. (**D**) DF-1 cells were co-transfected with Luc-YWHAZ 3′-UTR (wild-type or mutant) and the indicated RNA oligonucleotides. At 48 h post-transfection, the cells were assayed for both firefly and renilla luciferase activity through a dual-luciferase glow assay. Three independent experiments, each with three replicates, were performed. The data are expressed as the means ± SDs. One-way ANOVA was used to analyze the significant differences (Different lowercase letters represent *p* < 0.01).

To decipher the regulatory role of gga-miR-451 in MG-HS infection and to further investigate its potential molecular mechanism, we aimed to identify the exact target of gga-miR-451. We used the publicly available prediction software/servers from miRBase, miRDB, TargetScan, RNAhybrid, and miRecords to identify its targets possibly involved in the regulation of MG infection. The potential target YWHAZ was singled out for further study because it contains a miR-451 seed match in its 3′ UTR, and its encoded protein participates in the regulation of a variety of inflammatory cytokine production and cell cycle [26,32]. The sequence of target site in the 3′-UTR of YWHAZ is shown to be highly conserved among different species (Figure 3A). RNAhybrid was used to analyze the duplex and minimum free energy (mfe) between gga-miR-451 and the YWHAZ 3′-UTR. The mfe of the RNA duplex (−15.2 kCal/mol) indicated high stability (Figure 3B).

Furthermore, to determine whether YWHAZ is a direct and specific target of gga-miR-451 in DF-1 cells, we then constructed dual-luciferase reporter plasmids carrying the YWHAZ 3′-UTR with the wild-type or base pair mutant gga-miR-451 binding region to generate Luc-WT-YWHAZ (3′-UTR) and Luc-Mut-YWHAZ (3′-UTR) (Figure 3C). The luciferase activity significantly decreased when DF-1 cells were co-transfected with gga-miR-451 mimic and a wild-type YWHAZ (3′-UTR) luciferase reporter after 48 h. To confirm that this reduction in luciferase activity was indeed due to the interaction of gga-miR-451 with the 3′-UTR of YWHAZ, a mutant dual luciferase reporter containing four base pair mutations in the seed region was also co-transfected into DF-1 cells, together with miR-451-M or miR-451-NC. In addition, a mock transfection was used as a blank. As expected, no significant effect of either miR-451-M or miR-451-NC was observed (Figure 3D). These results demonstrate that gga-miR-451 negatively regulates the expression of YWHAZ by binding to the complementary sequence in the 3′-UTR of YWHAZ.

### 2.4. gga-miR-451 Negatively Regulates YWHAZ Expression

**Figure 4:**
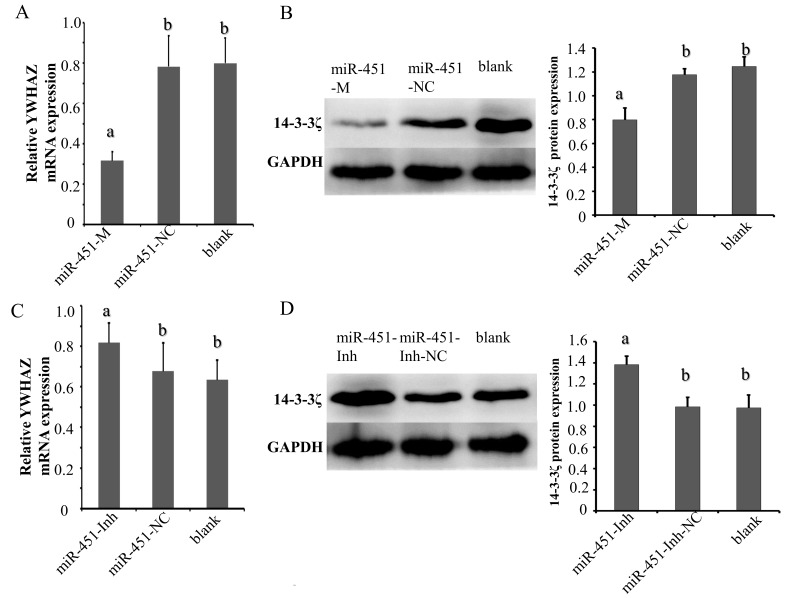
gga-miR-451 negatively regulates YWHAZ expression. DF-1 cells were transfected with the indicated RNA oligonucleotides. At 48 h post-transfection, RT-qPCR detected the expression of the mRNA level of YWHAZ, Western blot analysis of YWHAZ protein (14-3-3ζ) expression. (**A**) The mRNA level of YWHAZ in DF-1 cells over-expressing gga-miR-451 is reduced. (**B**) The protein level of YWHAZ (14-3-3ζ) in DF-1 cells over-expressing gga-miR-451 is reduced. (**C**) Transfection with miR-451-Inh increases the mRNA expression level of YWHAZ in DF-1 cells. (**D**) Transfection with miR-451-Inh increases the protein expression of YWHAZ (14-3-3ζ) in DF-1 cells. A mock transfection was used as a blank, and the expression of GAPDH served as a loading control. All of the error bars represent the means ± SDs of triplicate experiments. Three independent experiments, each with three replicates, were performed. One-way ANOVA was used to analyze the significant differences (Different lowercase letters represent *p* < 0.01).

To fully validate the impact of interaction between gga-miR-451 and the YWHAZ 3′-UTR, we tested the effects of the over-expression or suppression of gga-miR-451 on YWHAZ endogenous expression in DF-1 cells. Through transient transfection, the over-expression of gga-miR-451 markedly increased gga-miR-451 expression in DF-1 cells at 48 h post-transfection (Figure 2A). In addition, a mock transfection was used as a blank. The over-expression of miR-451-M, but not miR-451-NC, resulted in a significant reduction in YWHAZ (14-3-3ζ) at both the mRNA and protein levels (Figure 4A,B). The over-expression of miR-451-Inh markedly inhibited gga-miR-451 expression (Figure 2E), which restored the expression of YWHAZ (14-3-3ζ) at both the mRNA and protein levels (Figure 4C,D). The data suggest that YWHAZ is a direct target of gga-miR-451 and gga-miR-451 negatively regulates the expression of YWHAZ in DF-1 cells.

### 2.5. MG Infection Downregulates YWHAZ Expression

**Figure 5:**
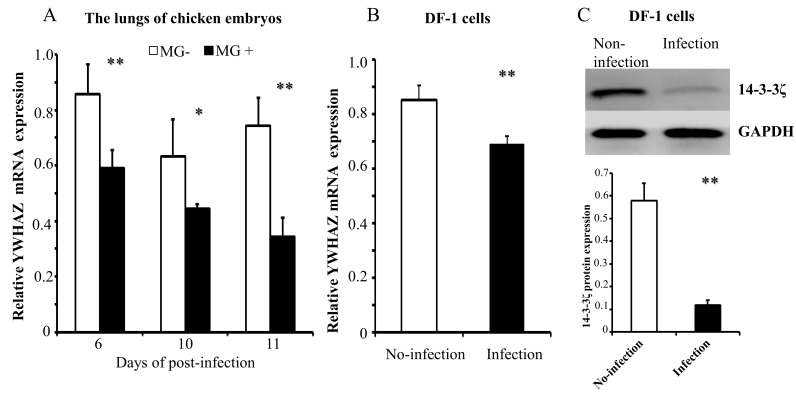
MG infection downregulates the expression of YWHAZ in chicken embryo lungs (**A**) and DF-1 cells (**B**). Chicken embryos and DF-1 cells were infected with MG-HS as described in Materials and Methods, and the total RNA was extracted. YWHAZ mRNA expression was assessed by RT-qPCR normalized to GAPDH. YWHAZ protein (14-3-3ζ) expression was determined by Western blotting and normalized to GAPDH (**C**). Three independent experiments, each with three replicates, were performed. Student *t* test. The plotted data points show the means ± SDs, and the asterisks indicate statistically significant differences (* *p* < 0.05, ** *p* < 0.01).

Having shown that MG infection upregulates gga-miR-451 expression and that YWHAZ is the direct target of gga-miR-451. To further verify the role of YWHAZ in MG-HS infection, we detected the expression of YWHAZ in MG-HS-infected chicken embryos and DF-1 cells. Chicken embryos were infected with MG-HS on the ninth day of incubation. On days 6, 10, and 11 post-infection (equivalent to days 15, 19, and 20 of egg incubation), qRT-PCR showed that the YWHAZ expression levels were significantly lower in the lungs of the MG-infected chicken embryos compared with the control (Figure 5A). As expected, the mRNA and protein levels of YWHAZ were both significantly down-regulated in MG-HS-infected DF-1 cells after 48 h detected by RT-qPCR and Western blot analysis separately (Figure 5B,C).

### 2.6. gga-miR-451 Inhibits Cell Proliferation by Affecting the Cell Cycle and Cell Apoptosis

**Figure 6:**
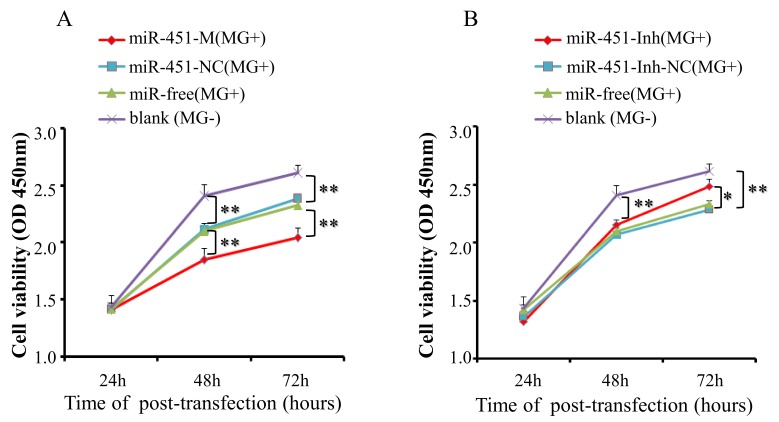
gga-miR-451 inhibits DF-1 cell proliferation in MG-HS infection. (**A**) The over-expression of gga-miR-451 remarkable inhibits DF-1 cell proliferation; (**B**) Inhibitor of gga-miR-451 promotes the proliferation of DF-1 cells. DF-1 cells were transfected with miR-451-M, miR-451-NC, miR-451-Inh, or miR-451-Inh-NC and were incubated for 24 h. The cells were then infected with the 8 µL of MG-HS strain. Four control groups, including miR-451-NC (MG+), miR-451-Inh-NC (MG+), miR-free (MG+), and the blank (MG−), were used. At 24, 48, and 72 h post-transfection, cell proliferation was detected using CCK-Cell Counting Kit-8. All values are represented as the mean ± SD of three independent experiments in triplicate. The asterisks represented statistically significant differences (* *p* < 0.05, ** *p* < 0.01).

To explore the potential functions of gga-miR-451 in MG-HS infection, DF-1 cells were transfected with miR-451-M or miR-451-NC and then co-cultured with MG-HS (8 µL at 10^10^ CCU/mL) for various periods of time (24 h, 48 h, and 72 h). In addition, DF-1 cells infected only with MG-HS (denoted as miR-free (MG+)), and DF-1cells neither transfected with miRNA nor infected with MG-HS (denoted as blank (MG−)) were used as control groups. The cell growth assay, using a Cell Counting Kit-8, showed that a decrease in cell viability in all MG-infected groups (including over-expression of miR-451-M, miR-451-NC, and miR-free) at 48 and 72 h post-transfection compared to the blank MG-group, the over-expression of miR-451-M significantly reduced the proliferation of DF-1 cells at 48 h and 72 h post-transfection compared with miR-451-NC and the miR-free control (Figure 6A). Furthermore, we studied the effects of miR-451 inhibitor on cell proliferation using the same grouping. After miR-451 inhibitor was transfected into DF-1 cells and co-cultured with MG-HS, this change resulted in a remarkable increase in the proliferation of DF-1 cells at 72 h post-transfection, compared to the miR-451-Inh-NC (MG+) group or the miR-free group (MG+) (Figure 6B). These results suggest that gga-miR-451 inhibits DF-1 cell proliferation in MG-HS infection.

**Figure 7:**
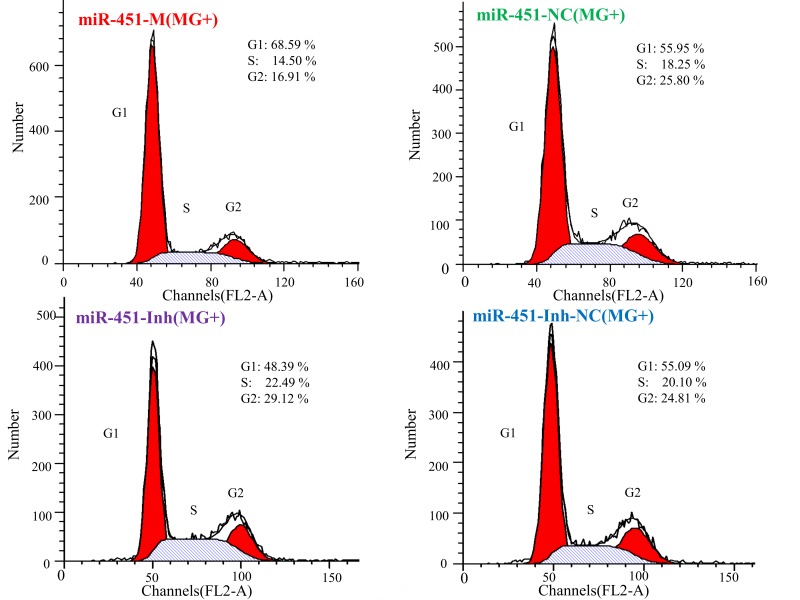

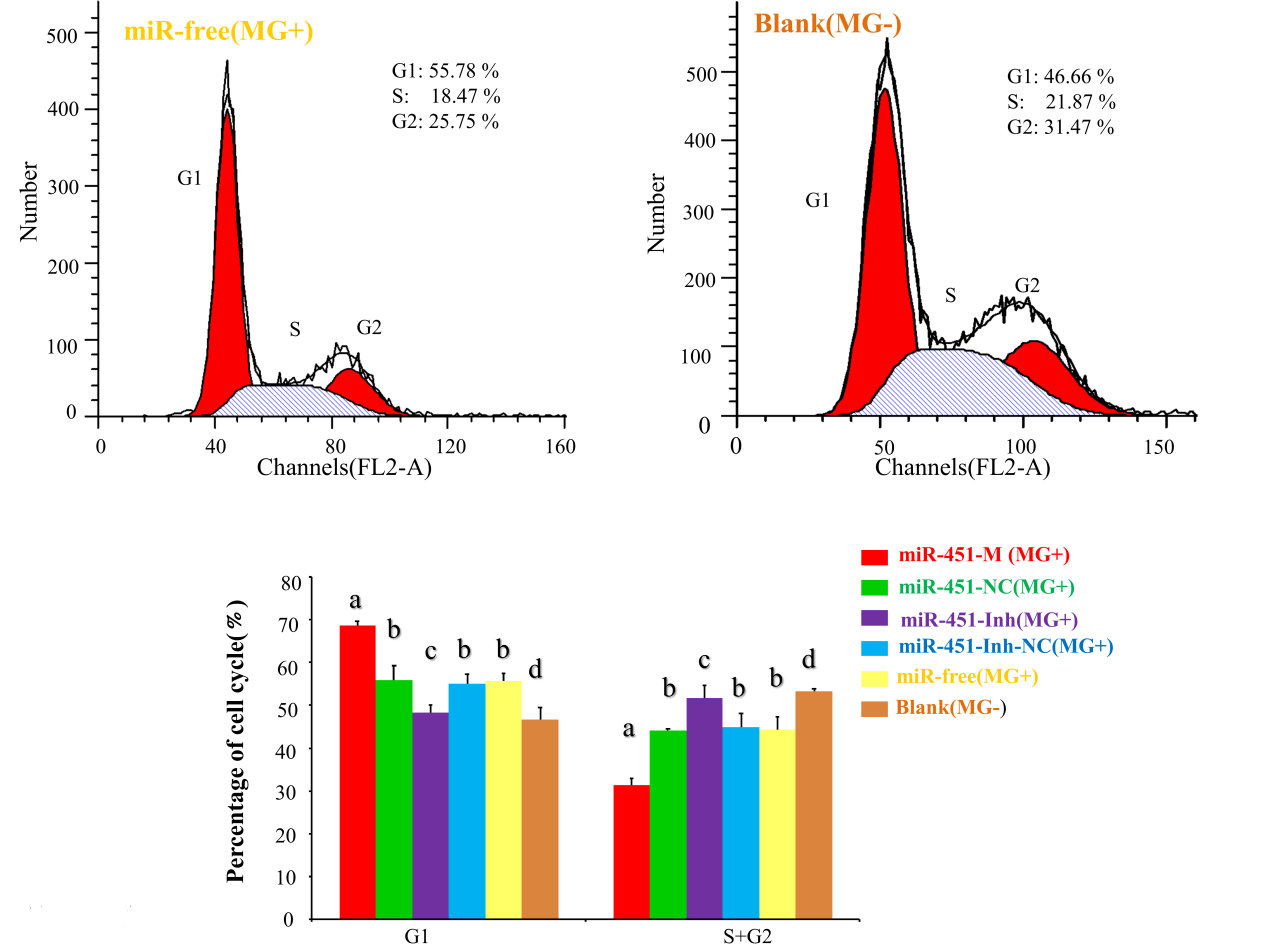
gga-miR-451 maintains DF-1 cells at the G1 phase in MG-HS infection. DF-1 cells were transfected with miR-451-M, miR-451-NC, miR-451-Inh, or miR-451-Inh-NC and were incubated for 24 h. The cells then were infected with MG-HS strain. Four control groups, including miR-451-NC (MG+), miR-451-Inh-NC (MG+), miR-free (MG+), and blank (MG−), were used. At 24 h post-infection, the cell phase distribution was analyzed using a flow cytometer. The FL2-A channel (*x* axis) shows the DNA number. Three independent experiments, each with three replicates, were performed. The data are presented as the means ± SDs. One-way ANOVA was used to analyze significant differences (Different lowercase letters represent *p* < 0.01 in G1 and S + G2 phase, respectively).

**Figure 8:**
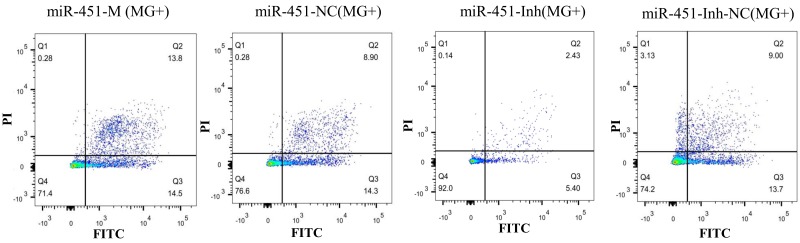

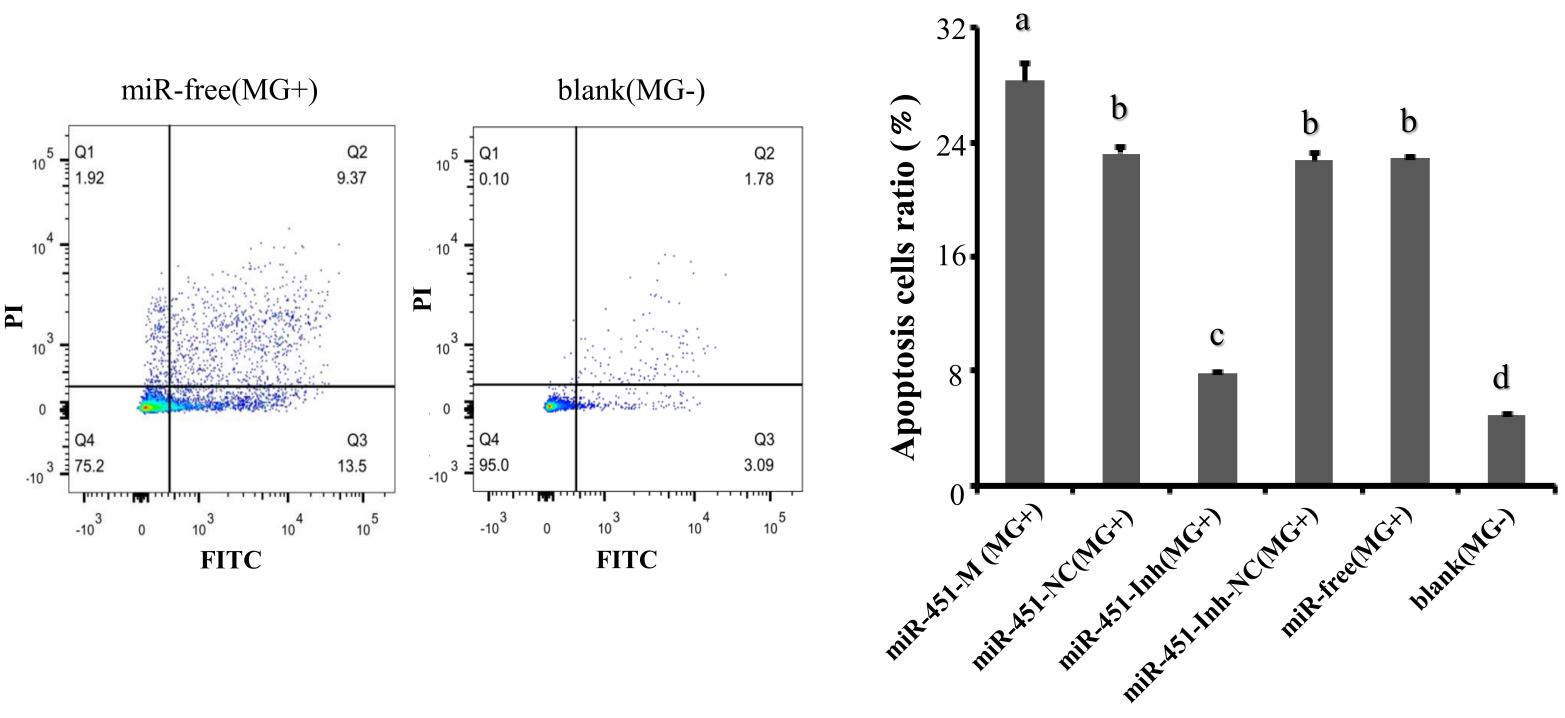
gga-miR-451 promotes DF-1 apoptosis in MG-HS infection. DF-1 cells were transfected with miR-451-M, miR-451-NC, miR-451-Inh, or miR-451-Inh-NC and were incubated for 24 h. The cells then were infected with MG-HS strain, harvested and stained with anti-annexin V-propidium iodide, and analyzed by flow cytometer after 24 h post-infection. Four control groups, including miR-451-NC (MG+), miR-451-Inh-NC (MG+), miR-free (MG+), and blank (MG−), were used. Three independent experiments, each with three replicates, were performed. The data are presented as the means ± SDs. One-way ANOVA was used to analyze significant differences (Different lowercase letters represent *p* < 0.01).

To further illustrate how gga-miR-451 inhibits DF-1 cells proliferation, we detected the effect of gga-miR-451 on the cell cycle distribution and cell apoptosis by flow cytometry. As above, the synthetic RNA oligonucleotides were transfected into DF-1 cells and then co-cultured with MG-HS (130 µL at 10^10^ CCU/mL) for 24 h. We demonstrated that MG infection inhibited mitosis by inducing the G1 cell cycle arrest in the DF-1 cells, overexpression of gga-miR-451 generated cell cycle changes with a larger proportion of cells in the G1 phase and a smaller proportion of cells in the S and G2 phases compared with miR-451-NC and the miR-free control. On the contrary, gga-miR-451-Inh promoted the cell cycle progression at the G1 phase compared with miR-451-Inh-NC and the miR-free control (Figure 7). Cell apoptosis assay showed that gga-miR-451 has a role in stimulating apoptosis of DF-1 cells. The percentage of apoptotic DF-1 cells increased when gga-miR-451 was overexpressed compared with miR-451-NC and the miR-free control, the opposite results were observed when miR-451 was silenced (Figure 8). Taken together, these results indicate that gga-miR-451 inhibits the proliferation of DF-1 cells by suppressing the cell cycle progression and increasing cell apoptosis.

## 3. Discussion

Innate/intrinsic host defenses are an imperative component of early and natural defenses against pathogenic microorganisms infection. However, evidence has shown that pathogen might evade host innate immunity through highly sophisticated mechanisms [33], and they can largely live and propagate in the host via suppressing host defence [34]. miRNAs have emerged as an important class of post-transcriptional regulators participated in the immune response and their regulatory features that may be key mechanisms in the control of microbial replication and homeostasis of the host innate response [35,36]. A large amount of evidence has demonstrated that miRNAs are involved in cellular behaviors with the aim of resisting or promoting inflammation during infections and inflammation in mammals [36,37]. Recently, we also reported the effects of gga-miR-19a, gga-miR-99a, and gga-miR-101-3p in MG-mediated inflammation in chicken [24,25,38]. However, the role of miRNAs in the MG-induced respiratory inflammation is largely unknown.

Evolutionary clustering showed that miR-451 is highly conserved in vertebrate evolution, indicating its functional importance in a wide range of species. miR-451 had been found to be ubiquitously expressed in different tissues [39,40]. Previous studies have suggested that miR-451 function in multiple cellular processes such as migration, invasion, cycle, proliferation, apoptosis, and it is an important and potential target in human cancer treatment [30,41,42,43]. However, little is known about the function and mechanism of miR-451 involving in MG-induced inflammation development and progression. In this study, we found that upregulation of miR-451 led to decrease the MG-induced production of inflammatory cytokines, inhibit the proliferation, cause cell cycle arrest and facilitate apoptosis by targeting YWHAZ. Our work explicitly indicated that MG-mediated miR-451 acted as a potent anti-inflammatory miRNA in the development of MG infection.

Our results show that gga-miR-451 was up-regulated in the lung tissues of MG-infected chicken embryos (on days 6, 10, and 11 post-infection) and in MG-infected DF-1 cells by RT-qPCR, but our previous deep sequencing results (on days 3 and 10 post-infection) showed the reverse [31]. The possible reason is that deep sequencing technology is suited for small RNA discovery with its high sensitivity, but small sample sizes of a single pool included six chicken embryos led to the decrease of collected reads, the deep sequencing results were influenced by random factors and biased with some errors [44]. In comparison, the experimental results are more credible, gga-miR-451 was highly expressed following MG infection. Similar to protein-coding genes, miRNA genes themselves are subject to sophisticated control [45]. Detailed mechanism involved in the activation of gga-miR-451 triggered by MG infection can provide insight for future studies.

*Mycoplasma* infects hosts by using adhesins to bind to host cells, which causes severe inflammation in humans and animals, and then induces a series of pro-inflammatory cytokines, apoptosis, or necrosis in a variety of cell types [46,47,48,49]. In DF-1 cells, MG infection stimulated the IL2/IL6-mediated inflammatory responses through TLR6-MyD88-NF-κB pathway [10]. In the lungs of chicken embryos, MG infection resulted in a significant increase of NF-κB and TNF-α expression [24]. Currently, TNF-α, IL-1β, and IL-6 inflammatory factors are usually responsible for the systemic effects of inflammation. Many studies have shown that TNF-α expression is associated with several diseases, including CRD, rheumatoid arthritis, pneumonia, and various forms of inflammation in birds and mammals [50]. Moreover, IL-1β and IL-6 participate in the occurrence and development of the disease by inducing extensive chemokine expression and upregulating adhesion molecules in human endothelial cells [51]. These results suggest that proinflammatory cytokines, such as TNF-α, IL-1β, and IL-6, play a key role in the development or progression of such inflammatory diseases by promoting inflammation and tissue injury. Remarkably, we observed that over-expression of gga-miR-451 could repress the production of inflammatory cytokines TNF-α, IL-1β, and IL6 in MG-infected DF-1 cells, inhibition of gga-miR-451 has the opposite effect. MG groups had higher levels of TNF-α, IL-1B, and IL-6 compared to no-infection groups (both miR-451-M and miR-451-Inh), as a result of the higher gga-miR-451 expression in the MG group. In Palmitate-exposed HepG2 cells, miR-451 expression negatively regulates inflammatory cytokine IL-8 and TNF-α expressions [52]. During viral infection, inhibition of miR-451 expression elevates secretion of IL-6 and TNF-α in three types of primary dendritic cells [26]. miR-451 inhibits inflammatory cytokines secretion of TNF-α, IL-1β, and IL6 in human rheumatoid arthritis [53]. It was consistent with our results. Our research proved that miR-451 could down-regulate corresponding cytokines levels and potentially improve the disease clinical manifestation.

miR-451 has been verified in multiple systems to directly bind YWHAZ and negatively regulates the expression of the YWHAZ by binding to a complementary sequence in the 3′-UTR of YWHAZ in mammals [27,28,29,30]. We also identified YWHAZ as a target of gga-miR-451 through a luciferase reporter assay, RT-qPCR, and Western blot analysis. The YWHAZ protein (14-3-3ζ) family is highly conserved and ubiquitously expressed in all eukaryotic organisms. YWHAZ (14-3-3ζ) is a member of the family of seven 14-3-3 proteins, all encoded by different genes [54]. YWHAZ serves as a pivotal factor that binds and stabilizes key proteins involved in cell proliferation, apoptosis, and signal transduction, including PKC, RAF-1, EGFR, HER2, and β-catenin [55]. YWHAZ is also known negative regulator of ZFP36, an RNA binding protein that targets AU-rich mRNAs such as CCL3, TNF-α, and IL-6 for degradation [26]. Decreasing YWHAZ levels by genetic dyrexpression or gga-miR-451 treatment correlated with increased ZFP36 levels a transcription factor that interacts with the transcriptional machinery to negatively regulate transcription of cytokines such as IL-6 and TNF-α [56,57]. Thus, it is reasonable to believe that MG infection upregulates gga-miR-451 expressions, gga-miR-451 targets the 3′-UTR of YWHAZ to decrease protein levels. YWHAZ might bind ZFP36 to decrease binding to AU-rich elements in the 3′-UTR of the proinflammatory cytokines TNF-α, IL-1β, and IL6, but the exact mechanism of this effect need to be further investigated.

Different expression of miRNAs plays key roles in regulating cell activities, including proliferation, cycle, morphogenesis, apoptosis, and differentiation. Moreover, we found that the over-expression of gga-miR-451 markedly suppressed MG-infected DF-1 cell proliferation, reduced the percentages of cells in the S and G2 phases of the cell cycle and promoted cell apoptosis. Previous studies have also reported that overexpression of miR-451 in QGY-7703, Hep3B, and MCF-7 cells greatly reduces YWHAZ expression and inhibits the cell proliferation [27]. miR-451 is identified to control the regulation of glioblastoma cell proliferation, invasion and apoptosis through the PI3K/AKT signaling pathway [58]. It is evident from the literature that miR-451 functions as a tumor suppressor and its down-regulation significantly reduces levels of YWHAZ and inhibits the cell proliferation [42,43], which further supports our results that gga-miR-451 decreasing YWHAZ expression contributes to the decreased cell proliferation during MG infection. YWHAZ protein has been shown to be key regulator of a large number of processes, such as control of regulation of human metabolism, cell cycle, cell proliferation, and apoptosis in multiple cells, and it promotes cell survival by association with proapoptotic proteins [59]. Thus, our findings suggest the possibility that decreased expression of YWHAZ mediated by gga-miR-451 up-regulation is responsible for the decreased cell proliferation and promoted apoptotic response associated with MG infection.

In conclusion, this study strongly suggests that the up-regulation of gga-miR-451 decreases YWHAZ expression in MG-infected tissues and cells, which reduces secretion of inflammatory cytokines and inhibits the cell cycle progression and cell proliferation, and promotes cell apoptosis to facilitate MG replication. These results provide evidence for anti-inflammatory effects of miR-451 which is mediated by targeting YWHAZ in MG infection. Furthermore, miR-451 and YWHAZ might be used as potential diagnostic biomarkers and therapeutic targets in the prevention and treatment of MG infection.

## 4. Materials and Methods

### 4.1. MG-HS Culture

MG-HS strain, isolated previously from a chicken farm in Hubei Province of China [12,13], was donated by the State Key Laboratory of Agricultural Microbiology, College of Veterinary Medicine, Huazhong Agricultural University (Wuhan, China). MG-HS was cultured at 37 °C in modified FM-4 medium supplemented with 12% (*v*/*v*) porcine serum and 10% yeast extract until the mid-log phase. The concentration of MG-HS was determined by the acid-mediated shift of phenol red dye from red to orange as previously described [12]. The number of viable *Mycoplasmas* in a suspension was then determined by a color-changing unit (CCU) assay [60].

### 4.2. Infection Experiments

One hundred embryos of White Leghorn specific-pathogen-free (SPF) chickens were incubated on the ninth day and the allantoic cavities were injected with 300 μL of MG-HS at 10^10^ CCU/mL. Other 100 chicken embryos were injected with the same dosage of the diluent to serve as controls. The viability of the chicken embryos was examined by eye under a candling machine. The dead embryos were eliminated. The mortality rates of the chicken embryos of the infection and control groups were 12.3% and 7%, respectively. Whole-lung tissue samples from six infected live chicken embryos and six controls were collected on days 6, 10, and 11 post-infection and stored in RNA fixer (BioTeke Co., Ltd., Beijing, China).

### 4.3. Cell Culture and Treatment

The chicken embryonic fibroblast cell line (DF-1), obtained from the American Type Culture Collection, was cultured in Dulbecco’s modified Eagle medium (DMEM; Gibco, Carlsbad, CA, USA) supplemented with 100 U/mL penicillin, 100 μg/mL streptomycin, and 10% fetal bovine serum (FBS; Invitrogen, Carlsbad, CA, USA) at 39 °C and 5% CO_2_. DF-1 cells were plated evenly in 6-, 24- or 96-well plates and grown to 60% confluency without antibiotics. Cells were subsequently transfected with RNAs, plasmids, or both, using Lipofectamine 3000 (Invitrogen). After 48 h, the cells in different groups were collected for further use. For MG-HS infection experiments, at 24 h post-transfection, cells were infected with MG-HS at the mid-exponential phase (1 × 10^10^ CCU/mL) for the times mentioned in the figure legends.

### 4.4. microRNA Target Prediction and Sequences

Putative gga-miR-451 target genes were identified using a miRNA database (http://www.mirbase.org/) and target prediction tools: miRDB (http://www.mirdb.org/miRDB/), PicTar (http://pictar.mdc-berlin.de/), and TargetScan (http://www.targetscan.org/). The conservation of the target gene was analyzed by TargetScan (http://www.targetscan.org/). The duplex and mfe between gga-miR-451 and the 3′-UTR of the potential targets were analyzed by RNAhybrid (https://bibiserv.cebitec.uni-bielefeld.de/rnahybrid/). AmiGO (http://amigo.geneontology.org) was used to analyze the functions of the target genes of gga-miR-451 in *Gallus gallus*.

The sequences of all of the primers used in this study are shown in Table 1. All RNA oligonucleotides were designed and synthesized by GenePharm (Shanghai, China) and are shown in Table 2.

### 4.5. Constructs and Plasmids

The psi-CHECK™-2 dual-luciferase reporter vector (Promega, Madison, WI, USA) harboring the wild-type and mutant YWHAZ 3′-UTR, which were inserted into the *Xho* I and *Not* I restriction sites 3′ to the end of the *Renilla* gene, were used to check the effect of gga-miR-451 on *Renilla* activity. The full length of the wild-type YWHAZ 3′-UTR or fragments covering the putative gga-miR-451-binding site were amplified by RT-PCR following cDNA extraction from the lung tissues of chicken. The psi-CHECK™-2 mutant YWHAZ 3′-UTR construct was generated by inducing a point mutation using the overlap extension PCR method. The recombinant wild-type and mutant plasmids were named Luc-WT-YWHAZ and Luc-Mut-YWHAZ, respectively. The primers are listed in Table 1. All constructs were verified by sequencing.

### 4.6. Dual-Luciferase Reporter Assay

DF-1 cells in 24-well plates were co-transfected with 200 ng of psiCHECK-2–YWHAZ-3′UTR (wild-type and mutant) and 10 pmol of the indicated RNA oligonucleotides using Lipofectamine 3000 (Invitrogen). The cells were collected at 48 h post-transfection, and the dual-luciferase activity assay was performed according to the manufacturer’s instructions (Promega). Luciferase activity was detected using a Lumat LB 9507 Ultra-Sensitive Tube Luminometer (Titertek Berthold, Nanjing, China). All transfection experiments were performed in triplicate and repeated at least three times.

### 4.7. RNA Extraction and Quantitative Real-Time (RT-qPCR)

Total RNA was extracted from the cultured cells and frozen lung tissue specimens of chicken embryos using the TRIzol reagent (Invitrogen). According to the manufacturer’s instructions, 1 μg RNA from each sample was used to synthesize cDNA using the Prime Script™ RT reagent kit with gDNA Eraser (TaKaRa, Tokyo, Japan). The real-time PCR was performed with TransStart Top Green qPCR SuperMix (TRANSGEN, Beijing, China) on a CFX96 or CFX384 Touch™ instrument (Bio-Rad, Hercules, CA, USA). The relative mRNA levels were calculated using the 2^−ΔΔ*C*t^ method [61]. The data were analyzed using 7500 software v.2.0.1 (Applied Biosystems, Foster City, CA, USA) with the automatic *C*t settings for adapting the baselines and thresholds for *C*t determination. The expression levels of gga-miR-451 and YWHAZ were measured by RT-qPCR. 5S-RNA and glyceraldehyde-3-phosphate dehydrogenase (GAPDH) mRNA were used as internal controls, respectively. The expression of 5S-RNA and GAPDH are stable in the course of MG infection. The experiment was repeated three times, and the primers are listed in Table 1.

### 4.8. Western Blot

The total proteins were isolated at 48 h post-transfection or post-infection using RIPA buffer (Beyotime, Nantong, China) supplemented with 100 mM phenylmethanesulfonyl fluoride (PMSF). Protein concentrations were measured with a BCA Protein Assay Kit (TRANSGEN), and 10 μg of protein was fractionated by 12% SDS-polyacrylamide gel electrophoresis (SDS-PAGE) and transferred by electrophoresis to polyvinylidene fluoride (PVDF) membranes (Beyotime, Nantong, China) using a Mini Trans-Blot Cell (Bio-Rad). The membranes were blocked in 5% (*w*/*v*) fat-free milk for 1 h at room temperature and incubated overnight with the appropriate primary antibodies, including rabbit polyclonal anti-YWHAZ (Cat. D155211-0025, Sangon Biotech, Shanghai, China) and *GAPDH* (Cat. 60004-1-1g, Proteintech, Wuhan, China), which served as the protein loading control. The membranes were then washed and incubated with goat anti-rabbit secondary antibody for 1.5 h. After washing with TBST, the proteins on the membranes were detected using an ECL™ detection system (Bio-Rad).

### 4.9. Electrophoretic Immunosorbent Assay (ELISA)

DF-1 cells in 6-well plates were transfected with miR-451-M, miR-451-NC, miR-451-Inh, or miR-451-Inh-NC and were incubated for 24 h and then either left uninfected or infected with 130 µL of MG-HS. The culture supernatants were collected from the treated cells at 36 h post-infection and stored at −80 °C. The protein levels of TNF-α, IL-1β, and IL6 in cell cultures were determined by Chicken enzyme-linked immunosorbent assay (ELISA) kits (Bio-Swamp, Shanghai, China) of TNF-α (Cat. CH50032), IL-1β (Cat. CH50020), and IL6 (Cat. CH50016) according to the manufacturer’s instructions.

### 4.10. Cell Proliferation, Cell Cycle and Cell Apoptosis

Cell proliferation was determined using the Cell Counting Kit-8 according to the manufacturer’s protocol (CCK-8, DOJINDO, Shanghai, China). DF-1 cells were plated at a density of 8 × 10^3^ cells/well in a flat-bottomed, 96-well cell culture plate and allowed to grow for 4 h at 39 °C with 5% CO_2_. The DF-1 cells were then transfected with gga-miR-451, miR-451-NC, miR-451-Inh, or miR-451-Inh-NC. At 24 h post-transfection, the cells then were infected with 8 µL of MG-HS strain at 10^10^ CCU/mL. At 24 h, 48 h, and 72 h post-transfection, 10 µL of the CCK-8 solution was added to each well of the plate, which was then incubated at 39 °C for 4 h. The infected-MG cells (denoted as miR-free MG+) and the uninfected-MG cells (blank MG−) were used as controls. The blank MG−cells were cultured in a sterile incubator to avoid MG contamination. The optical density at 450 nm of each well plate was measured using a microplate reader (Bio-Rad, Hercules, CA, USA).

The cell cycle and cell apoptosis assay were performed in 6-well plates. The DF-1 cells were transfected with the indicated RNA oligonucleotides. At 24 h post-transfection, the cells were infected with 130 µL of MG-HS strain at 10^10^ CCU/mL. At 24 h post-infection, the cell cycle was analyzed with a flow cytometer, using the cell cycle detection kit (KeyGEN, Nanjing, China). Similarly, miR-free-MG+ and blank MG− were used as controls. The percentages of the cells in the G1, S, and G2 phases were calculated. The cell apoptosis was measured with a flow cytometer, according to the manufacturer’s protocol of the Annexin V-FITC Apoptosis Detection Kit (DOJINDO, Shanghai, China).

### 4.11. Statistical Analysis

SPSS software (SPSS 20.0, IBM, Armonk, NY, USA) was used for statistical analyses. All results are presented as the mean values ± SEM. Statistical significance was determined by using one-way ANOVA or Student’s *t*-test, and *p*-values of ≤0.01 or ≤0.05 were considered the statistically significant difference between groups.

### 4.12. The Experimental Design

**Figure 9:**
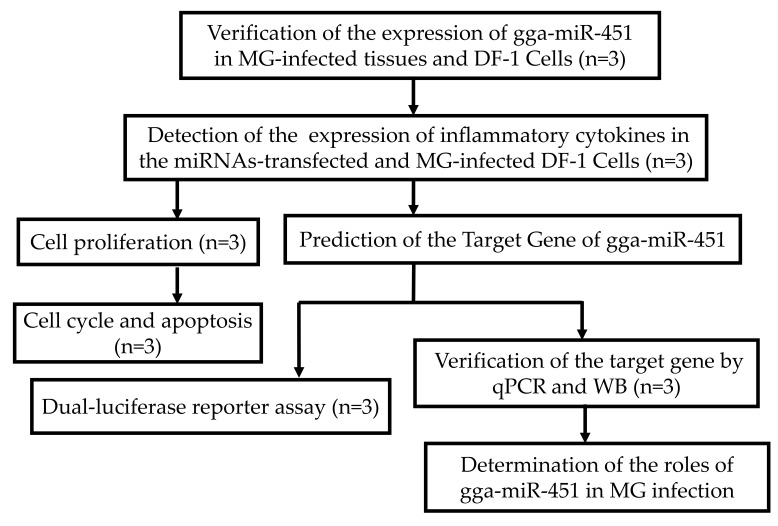
A graphic abstract showing the experimental design of this study (n represent biological replicates).

Firstly, the different expression of gga-miR-451 was verified in tissues and DF-1cells and the effect of gga-miR-451 was detected in the MG-HS-mediated inflammatory process. Then, the exact target of gga-miR-451 was identified. Finally, the potential functions of gga-miR-451 were investigated in MG-HS infection. Detailed experimental design is illustrated in Figure 9.

## Figures and Tables

**Table 1:** Sequences of DNA primers.

Name	Primer Sequence (5′-3′)	Accession No.
Primers for 3′-UTR Cloning		
YWHAZ 3′-UTR-F	GCGCTCGAGCAAGCGAGGAAGACT	NM_001031343.1
YWHAZ 3′-UTR-R	ATTGCGGCCGCAAGCAACAGCAGGTA	
Mut-P-Y 3′-UTR-F	GAAGGACAGAGAAAGTCATCACATTCCATTATTTGTAAAGTTACCTGCTGTTGC	NM_001031343.1
Mut-P-R 3′-UTR-R	AATAATGGAATGTGATGACTTTCTCTGTCCTTCTCTAGAGAAAGTTGACTGG	
Primers for RT-qPCR		
GAPDH-F	GAGGGTAGTGAAGGCTGCTG	NM 204305
GAPDH-R	CACAACACGGTTGCTGTATC	
YWHAZ-F	AAAATGTTGTAGGAGCCCGTAGG	NM_001031343.1
YWHAZ-R	TTGCTTTCTGCTTGCGAAGC	
RT-gga-miR-451	CTCAACTGGTGTCGTGGAGTCGGCAATTCAGTTGAGAAACTCAG	MIMAT0003775
gga-miR-451-F	GTAGGAAACCGTTACCATTACTGAG	MIMAT0003775
gga-miR-451-R	ACTGGTGTCGTGGAGTCGGC	
gga-5s-rRNA-F	CCATACCACCCTGGAAACGC	
gga-5s-rRNA-R	TACTAACCGAGCCCGACCCT	

**Table 2:** Sequences of RNA oligonucleotides.

Name	Sequences (5′-3′)
gga-miR-451 mimics	AAACCGUUACCAUUACUGAGUUUACUCAGUAAUGGUAACGGUUUUU
gga-miR-451 NC	UUCUCCGAACGUGUCACGUTTACGUGACACGUUCGGAGAATT
gga-miR-451 inhibitor	AAACUCAGUAAUGGUAACGGUUU
gga-miR-451 inhibitor NC	CAGUACUUUUGUGUAGUACAA
